# Pathogenicity Determinants of the Human Malaria Parasite *Plasmodium falciparum* Have Ancient Origins

**DOI:** 10.1128/mSphere.00348-16

**Published:** 2017-01-11

**Authors:** Andrew J. Brazier, Marion Avril, Maria Bernabeu, Maxwell Benjamin, Joseph D. Smith

**Affiliations:** a Center for Infectious Disease Research, Seattle, Washington, USA; b Department of Global Health, University of Washington, Seattle, Washington, USA; The Hebrew University

**Keywords:** *Plasmodium falciparum*, *Plasmodium reichenowi*, cytoadhesion, *var* gene

## Abstract

*Plasmodium falciparum*, the most deadly of the human malaria parasites, is a member of the *Laverania* subgenus that also infects African Great Apes. The virulence of *P. falciparum* is related to cytoadhesion of infected erythrocytes in microvasculature, but the origin of dangerous parasite adhesion traits is poorly understood. To investigate the evolutionary history of the *P. falciparum* cytoadhesion pathogenicity determinant, we studied adhesion domains from the chimpanzee malaria parasite *P. reichenowi*. We demonstrate that the *P. reichenowi var* gene repertoire encodes cysteine-rich interdomain region (CIDR) domains which bind human CD36 and endothelial protein C receptor (EPCR) with the same levels of affinity and at binding sites similar to those bound by *P. falciparum*. Moreover, *P. reichenowi* domains interfere with the protective function of the activated protein C-EPCR pathway on endothelial cells, a presumptive virulence trait in humans. These findings provide evidence for ancient evolutionary origins of two key cytoadhesion properties of *P. falciparum* that contribute to human infection and pathogenicity.

**IMPORTANCE** Cytoadhesion of *P. falciparum*-infected erythrocytes in the microcirculation is a major virulence determinant. *P. falciparum* is descended from a subgenus of parasites that also infect chimpanzees and gorillas and exhibits strict host species specificity. Despite their high genetic similarity to *P. falciparum*, it is unknown whether ape parasites encode adhesion properties similar to those of *P. falciparum* or are as virulent in their natural hosts. Consequently, it has been unclear when virulent adhesion traits arose in *P. falciparum* and how long they have been present in the parasite population. It is also unknown whether cytoadhesive interactions pose a barrier to cross-species transmission. We show that parasite domains from the chimpanzee malaria parasite *P. reichenowi* bind human receptors with specificity similar to that of *P. falciparum*. Our findings suggest that parasite adhesion traits associated with both mild and severe malaria have much earlier origins than previously appreciated and have important implications for virulence evolution in a major human pathogen.

## Abstract (precis)

Cytoadhesion of *P. falciparum*-infected erythrocytes in the microcirculation is a major virulence determinant. *P. falciparum* is descended from a subgenus of parasites that also infect chimpanzees and gorillas and exhibits strict host species specificity. Despite their high genetic similarity to *P. falciparum*, it is unknown whether ape parasites encode adhesion properties similar to those of *P. falciparum* or are as virulent in their natural hosts. Consequently, it has been unclear when virulent adhesion traits arose in *P. falciparum* and how long they have been present in the parasite population. It is also unknown whether cytoadhesive interactions pose a barrier to cross-species transmission. We show that parasite domains from the chimpanzee malaria parasite *P. reichenowi* bind human receptors with specificity similar to that of *P. falciparum*. Our findings suggest that parasite adhesion traits associated with both mild and severe malaria have much earlier origins than previously appreciated and have important implications for virulence evolution in a major human pathogen.

## INTRODUCTION

*Plasmodium falciparum* is the most deadly infective parasite in the world, with approximately 300 million clinical episodes and hundreds of thousands of deaths annually (1). The greater virulence of *P. falciparum* compared to other human malaria parasites is attributable to the ability of parasites to infect red blood cell stages of different ages, thereby contributing to a higher parasite biomass, and the unique capability of *P. falciparum*-infected erythrocytes (IEs) to sequester in the microcirculation (2). Cytoadhesion prevents the splenic elimination of IEs, but it can lead to vaso-occlusion, metabolic acidosis, and organ-specific disease complications (3–6). *P. falciparum* is distantly related to other human malaria parasites, and its closest relatives are parasites of gorillas and chimpanzees, termed the *Laverania* subgenus (7–11). Phylogenetic analysis suggests that *P. falciparum* was introduced to humans through zoonotic transfer of a gorilla parasite (*P. praefalciparum*) (8).

Cytoadhesion of falciparum malaria involves significant remodeling of the erythrocyte membrane cytoskeleton to form distinctive, knob-like protrusions (12–15). These modifications result in reductions in the deformability of IEs (16) and render them vulnerable to splenic clearance (17). Parasite binding to vascular endothelium is mediated by a clonally variant gene family, termed the *var* gene or *P. falciparum* erythrocyte membrane protein 1 (PfEMP1) family (18–20). PfEMP1 proteins contain multiple Duffy binding-like (DBL) and cysteine-rich interdomain region (CIDR) domains that interact with endothelial receptors (21). Surface exposure places PfEMP1 proteins under strong selection for immune evasion and binding properties, resulting in high intra- and interstrain sequence variability (22). Nevertheless, the *var* gene family is organized similarly between parasite genotypes into three major types (A, B, and C) and a placenta-specific E variant, as defined by the chromosomal location and 5′ upstream sequence region (23–25). Furthermore, *var* groups have diverged into CD36 binding (groups B and C) and endothelial protein C receptor (EPCR) binding (group A) subsets, with both traits mapping to the CIDR domain in the PfEMP1 head structure (26–29). Infections dominated by CD36 binding parasites are associated with mild malaria, while parasites transcribing *var* genes that are predicted to encode EPCR binding properties are preferentially expressed in malaria-naive hosts and in subjects with severe malaria (29–36). EPCR plays an important role in regulating coagulation, vascular inflammation, and endothelial permeability (37), and it is thought that parasite blockade of EPCR function may contribute to malaria disease mechanisms (30, 38–40). Both the conservation of *var* gene genomic organization and protein functional specialization suggest that adhesion selection has strongly shaped the PfEMP1 repertoire, although some adhesion traits appear to be more dangerous than others.

Despite the importance of IE sequestration in virulence, the vast majority of *P. falciparum* infections do not lead to severe disease, suggesting that cytoadhesion is relatively well adapted. An approach to investigating the evolutionary history of pathogenicity determinants is to study other *Laverania* parasites. Although limited genetic data exist for most *Laverania* parasite species, the chimpanzee malaria parasite *P. reichenowi* has been fully sequenced and presents extensive gene synteny with *P. falciparum*, including multigene families involved in erythrocyte remodeling and a repertoire of *var* genes with similar gene copy numbers and multidomain architectures (41–44). By comparison, *P. gaboni*, a *Laverania* parasite more distantly related to *P. falciparum*, contains divergent *var*-like genes (41). Notably, *var* genes are absent in non-*Laverania* malaria species (45, 46), indicating that the *var*-mediated cytoadhesion phenotype originated within the *Laverania* subgenus.

The *var* gene/PfEMP1 family has a capacity for rapid evolution through high rates of recombination and mutation (47). These features endow the members of the protein family with a far greater versatility than ordinary malaria proteins with respect to escaping immunity and potentially acquiring new adhesion traits. Notably, minor sequence variation in the *P. reichenowi* and *P. falciparum* reticulocyte binding protein homologue 5 (RH5) invasion ligand has a major role in determining host tropism for red blood cells (48). However, it is unknown whether cytoadhesion interactions impose a similar host restriction barrier for cross-species transmission of ape *Laverania* parasites to humans. It is also not known when virulent adhesion traits arose in *P. falciparum*. Here, we performed the first functional characterization of domains from *P. reichenowi* erythrocyte membrane protein 1 (PrEMP1). We provide evidence that CD36 and EPCR head structure binding properties have ancient origins that predate *P. reichenowi* and *P. falciparum* speciation into chimpanzee and human hosts, thereby revealing deep evolutionary roots of parasite adhesion traits that have been linked to both mild and severe infection outcomes.

## RESULTS

### Sequence comparison of CIDR domains in *P. reichenowi* and *P. falciparum*.

**FIG 1:**
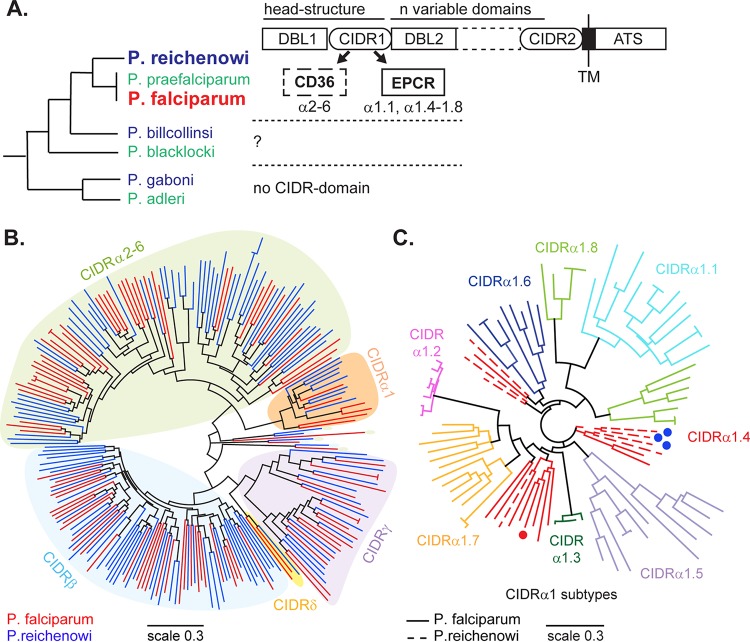
Phylogenetic analysis of *P. reichenowi* and *P. falciparum* CIDR sequences. (A) Protein schematic illustrating the multidomain architecture of PfEMP1 proteins. The CIDR1 domain in the PfEMP1 head structure has diversified into CD36 binding (CIDRα2-6) and EPCR binding (CIDRα1.1, CIDRα1.4-CIDRα1.8) sequences. The phylogenetic relationships of *Laverania* parasites are shown at the left (41). *P. falciparum* and *P. reichenowi* have similar DBL and CIDR multidomain PfEMP1 architectures (25, 42), *P. gaboni* has DBL domains but lacks CIDR domains (41), and the domain structure is unknown for other *Laverania* species. ATS, acidic terminal sequence; TM, transmembrane. (B) A neighbor joining tree was constructed from CIDR domains in the *P. reichenowi* CDC isolate (blue lines) and from the 3D7 genome reference isolate of *P. falciparum* (red lines). The major CIDR sequence types (α and β, γ and δ) are indicated by shading. (C) A neighbor joining tree of representative CIDRα1.1-1.8 subtype sequences from seven *P. falciparum* genotypes (25, 26, 30) (solid lines) and CIDRα1 sequences in *P. reichenowi* (dashed lines). The CIDR subtypes are labeled and indicated by coloring. Blue dots indicate the expressed *P. reichenowi* domains, and the red dot indicates the expressed *P. falciparum* domain.

CIDR domains in *P. falciparum* are classified into four major sequence types (α, β, γ, and δ), as well as CIDRpam in the placenta-specific VAR2CSA variant, on the basis of sequence similarity and phylogenetic classification (25, 49). Most PfEMP1 proteins contain two CIDR domains (Fig. 1A). The CIDR domain in the PfEMP1 head structure has diversified into CIDRα1 domains (EPCR binders), CIDRα2-6 domains (CD36 binders), and CIDR β, γ, and δ domains (unknown binding type) (Fig. 1A). The second CIDR domain located in the C terminus is always of the β, γ, or δ type, but it is unknown if it has receptor binding properties. Whereas the *var* genes in *P. reichenowi* and *P. falciparum* encode similar DBL and CIDR multidomain protein architectures (25, 42), the *var*-like genes of *P. gaboni* lack CIDR domains (41) (Fig. 1A). As an approach to investigation of the evolutionary history of the CIDR domain and of its sequence and functional specialization, we compared domains from *P. reichenowi* and *P. falciparum*.

In the phylogenetic tree, *P. falciparum* and *P. reichenowi* CIDR sequences were interspersed and did not cluster by species (Fig. 1B). Moreover, the four major types of CIDR domains (α, β, γ, and δ) were also present in the *P. reichenowi* genome (Fig. 1B). Like *P. falciparum*, predicted CD36 binding types were more common than EPCR binding types in *P. reichenowi* (Fig. 1B). Given the association of EPCR binding with severe malaria (29, 30), additional subtyping of *P. reichenowi* CIDRα1 domains was performed. Of the eight CIDRα1 subtypes in *P. falciparum* (25), six have been shown to encode EPCR binding activity (CIDRα1.1 and CIDRα1.4 to CIDRα1.8 [CIDRα1.4-1.8]) and two do not (CIDRα1.2 and 1.3 [CIDRα1.2-1.3]) (26). In the CIDRα1 subtype tree (Fig. 1C), all of the *P. reichenowi* CIDRα1 sequences clustered with CIDRα1.4 sequences from *P. falciparum*. Viewing the data as a whole, this analysis supports the conclusion that the divergence of CIDR sequence types occurred prior to speciation of *P. reichenowi* and *P. falciparum* and that the same major sequence types have been maintained during parasite adaptation to their chimpanzee and human hosts.

### Analysis of binding of *P. reichenowi* CIDR domains for CD36 or EPCR.

**FIG 2:**
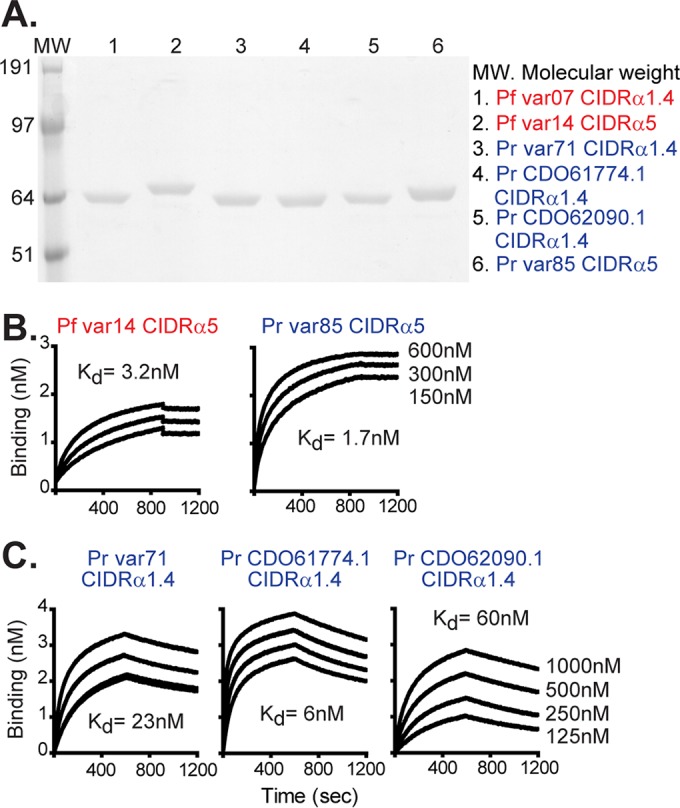
*P. reichenowi* CIDR domains bind to recombinant human CD36 and EPCR. (A) *P. falciparum* (Pf) and *P. reichenowi* (Pr) CIDR recombinant proteins were analyzed under reducing conditions in an SDS-PAGE gel and stained with GelCode Blue protein stain. (B and C) Sensorgrams of CIDR proteins binding to recombinant human CD36 (B) or recombinant human EPCR (C). *P. falciparum* domains are indicated with red font and *P. reichenowi* domains with blue font. The concentrations of recombinant CIDR domains analyzed in panels B and C are indicated to the right of the sensorgrams.

To investigate whether the CD36 and EPCR binding traits arose before or after *P. falciparum* crossed to humans, we expressed representative CIDR domains from *P. reichenowi*, including one domain that clustered with CD36 binding *P. falciparum* domains (*P. reichenowi* var85 CIDRα5) and three domains that clustered with EPCR binding *P. falciparum* domains (*P. reichenowi* var71 CIDRα1.4, *P. reichenowi* CD061774.1 CIDRα1.4, and *P. reichenowi* CDO62090.1 CIDRα1.4). For controls, a CD36 binding domain (*P. falciparum* var14 CIDRα5) and an EPCR binding domain (*P. falciparum* var07 CIDRα1.4) from *P. falciparum* strain IT4/25/4 were analyzed. All of the recombinant proteins ran as a single predominant band at the expected size on an SDS-PAGE gel (Fig. 2A). By size exclusion chromatography, the majority of *P. reichenowi* var85 CIDRα5 (67%) and *P. reichenowi* CD061774.1 CIDRα1.4 (60%) eluted at the expected monomeric size and *P. reichenowi* var71 CIDRα1.4 was 100% monomeric (see Fig. S1 in the supplemental material).

10.1128/mSphere.00348-16.1 FIG S1  Size exclusion chromatography (SEC) of *P. reichenowi* CIDR domains. Analytical SEC was performed in Superdex 200 columns for *P. reichenowi* (Pr) CDO61774.1 CIDRα1.4 (A), *P. reichenowi* var71 CIDRα1.4 (B), and *P. reichenowi* var85 CIDRα5 (C). The traces show the elution of the protein (UV absorbance), with protein standards in red and CIDR domains in blue. Download FIG S1, PDF file, 0.3 MB. Copyright © 2017 Brazier et al. 2017 Brazier et al. This content is distributed under the terms of the Creative Commons Attribution 4.0 International license.

Using biolayer interferometry (BLI), the *P. reichenowi* CIDR domains bound human CD36 (Fig. 2B) or human EPCR (Fig. 2C) in a manner predicted by their phylogenetic classification. Furthermore, the dissociation constants were similar to their counterparts in *P. falciparum*. For instance, the *P. reichenowi* and *P. falciparum* CIDR domains bound human CD36 with nearly identical affinities (for *P. falciparum* var14 CIDRα5, equilibrium dissociation constant [*K*_*d*_] = 3.2 nM; for *P. reichenowi* var85 CIDRα5, *K*_*d*_ = 1.7 nM). These *K*_*d*_ values are similar to binding constants reported for other CIDRα2-6 domains from *P. falciparum* (50). Likewise, the *P. reichenowi* CIDR domains bound EPCR with nanomolar affinity (for *P. reichenowi* var71 CIDRα1.4, *K*_*d*_ = 23 nM; for *P. reichenowi* CDO61774.1 CIDRα1.4, *K*_*d*_ = 6 nM; for *P. reichenowi* CDO62090.1 CIDRα1.4, *K*_*d*_ = 60 nM). By comparison, the *P. falciparum* var07 CIDRα1.4 domain has 2 nM affinity for EPCR (26, 40), and *K*_*d*_ values have been shown to range between 0.3 and 60 nM for other PfCIDRα1-EPCR interactions (26, 29, 30, 40). Moreover, the association rates of *P. reichenowi* CIDRα1 (upper 10^3^ to 10^4^) and the dissociation rates (10^−4^) were equivalent to those determined for PfCIDRα1-EPCR interactions (26, 29, 30, 40) (see Table S1 in the supplemental material). Although the presence of higher-molecular-weight species in the *P. reichenowi* var85 CIDRα5 and the *P. reichenowi* CD061774.1 CIDRα1.4 protein preparations may have affected the precision of the kinetic measurements for those two domains, the *P. reichenowi* var71 CIDRα1.4 protein was 100% monomeric (Fig. S1). Therefore, the chimpanzee malaria domains bound human receptors with kinetics highly similar to *P. falciparum* kinetics.

10.1128/mSphere.00348-16.2 TABLE S1  Affinities of CIDR domains for EPCR or CD36 as measured by biolayer interferometry (BLI). CIDR, cysteine-rich interdomain region; Kd, equilibrium dissociation constant; kon, association rate constant; koff, dissociation rate constant, Pr, *Plasmodium reichenowi*; Pf, *Plasmodium falciparum*; EPCR, endothelial protein C receptor; CD36. Download TABLE S1, PDF file, 0.02 MB. Copyright © 2017 Brazier et al. 2017 Brazier et al. This content is distributed under the terms of the Creative Commons Attribution 4.0 International license.

**FIG 3:**
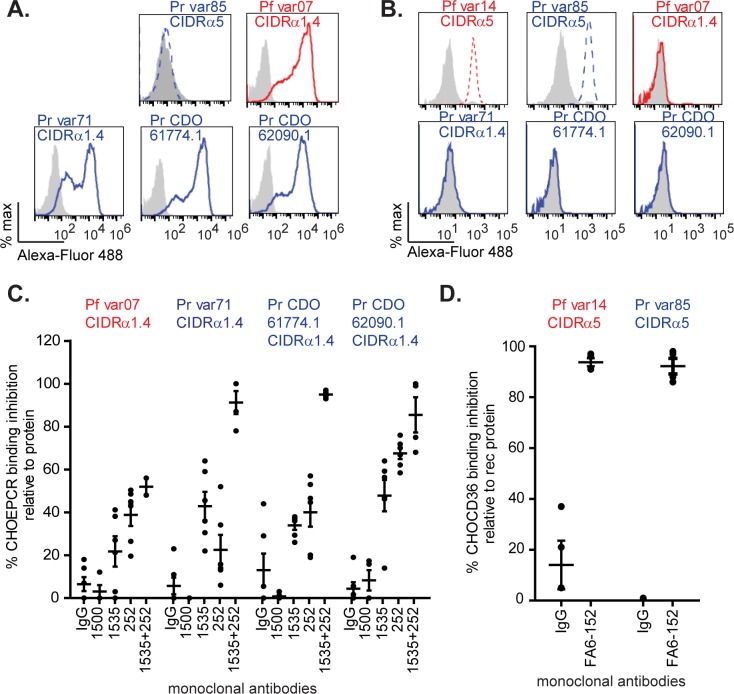
Binding of *P. reichenowi* CIDR domains to human CD36 and EPCR in cell-based assays. (A) Histograms show binding of recombinant CIDR domains to CHO745 untransfected cells (gray histogram) versus CHO745-EPCR cells (blue for *P. reichenowi*, red for *P. falciparum*, dashed line for CD36 binder, solid line for EPCR binder). (B) Dot blots with means (lines) show the percentages of inhibition of binding to CHO745-EPCR cells in the presence of different anti-EPCR monoclonal antibodies versus an isotype control antibody (means and standard errors of the means [SEM]; *n* = 4 to 6 from 2 to 3 independent experiments done in duplicate). (C) Histograms show binding of recombinant CIDR domains to CHO745 untransfected cells (gray histogram) versus CHO745-CD36 cells. (D) Dot blots with means (lines) show the percentages of inhibition of binding to CHO745-CD36 cells in the presence of anti-CD36 MAb FA6-152 versus an isotype control antibody (means and SEM; *n* = 4 from 2 independent experiments done in duplicate). Representative histograms from panels B and D are shown in Fig. S2.

Similarly, in cell binding assays, the *P. reichenowi* CIDR domains bound with predicted specificity to CHO-EPCR cells (Fig. 3A) or CHO-CD36 cells (Fig. 3B) transfected with human receptors but not to untransfected CHO745 cell lines. To further delineate the fine specificity of the *P. reichenowi* and *P. falciparum* domains for human receptors, anti-EPCR and anti-CD36 monoclonal antibodies (MAbs) were employed (Fig. 3C and D and Fig. S2). As previously reported, anti-EPCR MAbs differ in levels of blocking activity (40). Whereas the *P. falciparum* and *P. reichenowi* CIDR domains were partially inhibited by the anti-EPCR MAbs 1535 and 252 (mean, 22% to 68% reduction), the extents of inhibition in the species were similar (Fig. 3C). Furthermore, a combination of 1535 and 252 MAbs completely abolished binding of *P. reichenowi* domains to CHO-EPCR cells (Fig. 3C and Fig. S2). By comparison, there was no or little inhibition of CIDR domains with an isotype control antibody or nonblocking, anti-EPCR MAb 1500 (Fig. 3C) (40).

10.1128/mSphere.00348-16.3 FIG S2  Representative flow cytometry histograms of anti-EPCR and anti-CD36 antibody inhibition. (A) Histograms of recombinant CIDR domains binding to CHO745-EPCR cells in the presence or absence of anti-EPCR MAbs (rat MAb 252 or mouse MAb 1535 or 1500) or a rat IgG isotype control antibody for MAb 252. (B) Histograms of recombinant CIDR domains binding to CHO745-CD36 cells in the presence or absence of anti-CD36 MAb (mouse MAb FA6-152) or a mouse IgG isotype control antibody. Panels A and B show representative histograms from *n* = 4 replicates from 2 independent experiments. The gating strategy and methodology used for calculating the percentage of binding inhibition represented in Fig. 3C are shown in panel C. Download FIG S2, PDF file, 0.5 MB. Copyright © 2017 Brazier et al. 2017 Brazier et al. This content is distributed under the terms of the Creative Commons Attribution 4.0 International license.

For the CD36 interaction, both the *P. falciparum* and *P. reichenowi* domains were inhibited at levels greater than 90% by the anti-CD36 MAb FA6-152 and there was limited or no inhibition by an isotype control antibody (Fig. 3D and Fig. S2). A recent analysis of a CD36:CIDRα2.8 co-crystal structure identified 14 residues in CD36 that interact with Malayan Camp var1 CIDRα2.8 (50). Human and chimpanzee CD36 sequences are identical at 13 of 14 contact residues (conservative substitution I157V), and human and gorilla CD36 sequences are identical at 8 of 14 contact residues (conservative substitutions at M156V and I157V) (Fig. S3). Taken together, the results of this analysis indicate that CIDR domains from *P. reichenowi* and *P. falciparum* interact with similar regions on CD36 and EPCR and further suggest that these binding properties originated in a common ancestor of *P. reichenowi* and *P. falciparum*.

10.1128/mSphere.00348-16.4 FIG S3  Sequence comparison of CD36 from human, chimpanzee, and gorilla. A human CD36 sequence (GenBank accession no. NP_001001547.1) is shown at the top of the lineup. Residues that differ in chimpanzee (GenBank accession no. JAA08227.1) and gorilla (GenBank accession no. XP_004045702.1) sequences are indicated. CD36 residues that are directly contacted by the Malayan Camp var1 CIDRα2.8 (50) are indicated by the caret symbol. Download FIG S3, PDF file, 0.01 MB. Copyright © 2017 Brazier et al. 2017 Brazier et al. This content is distributed under the terms of the Creative Commons Attribution 4.0 International license.

### CIDR domains from *P. reichenowi* interfere with the endothelial barrier protective response of the EPCR pathway.

EPCR is a receptor for protein C/activated protein C (APC) and plays a critical role in coagulation, inflammation, and endothelial barrier properties (37). It has been postulated that EPCR binding *P. falciparum* parasites contribute to cerebral malaria brain swelling (51) by inhibiting the APC-EPCR interaction (29, 38–40). To explore the origins of this presumptive virulence phenotype in the *Laverania* subgenus, we investigated whether *P. reichenowi* CIDR domains inhibit the APC-EPCR interaction to the same extent as *P. falciparum* domains.

**FIG 4:**
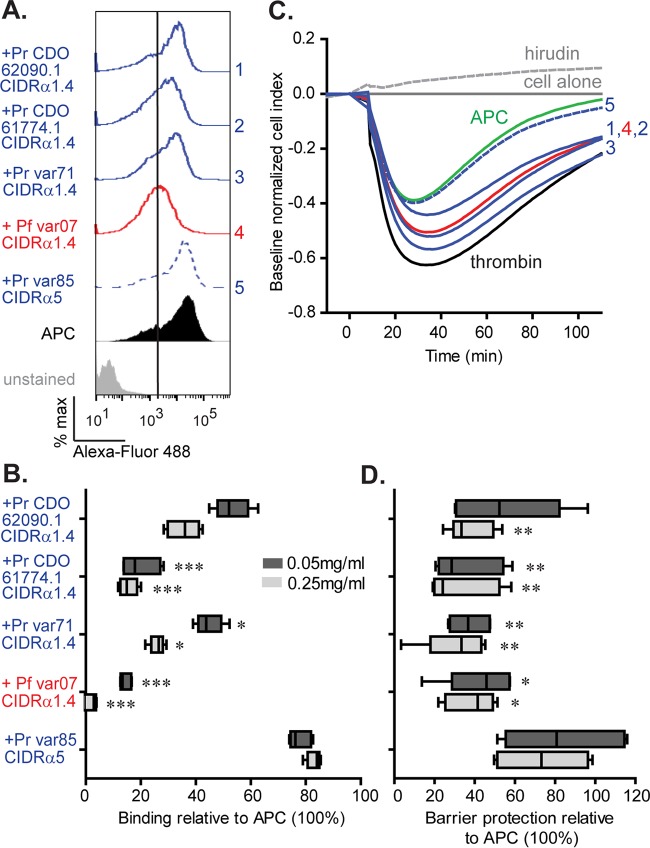
*P. reichenowi* CIDR domains inhibit the APC-EPCR interaction. (A) Level of APC binding alone (black histogram) versus binding in the presence of 250 μg/ml of recombinant CIDR domains to CHO745-EPCR cells (blue for *P. reichenowi*, red for *P. falciparum*, dashed line for CD36 binder, solid line for EPCR binder). The vertical line shows the secondary antibody alone used to set the background level. (B) Boxes with medians (lines in boxes) and whiskers (10th to 90th percentiles) show the percentages of APC binding in the presence of 50 μg/ml (dark gray) or 250 μg/ml (light gray) recombinant CIDR domains relative to APC binding alone (means and standard deviations; *n* = 3 independent experiments). *, *P* < 0.05; ***, *P* < 0.001 (by the nonparametric Kruskal-Wallis two-sided test followed by *post hoc* multiple comparisons performed using Dunn’s test). (C) Kinetics showing APC (100 nM, green line)-mediated protection of thrombin (5 nM, black line)-induced barrier disruption in primary human brain endothelial cell monolayers. The ability of CIDR domains to inhibit the barrier protective properties of APC was measured at the peak of thrombin-induced barrier disruption. The thrombin inhibitor hirudin (dotted gray line) was used as a control to block thrombin-induced permeability. The single digits by each curve refer to the labeled proteins in panel A. (D) Boxes with medians (lines in boxes) and whiskers (10th to 90th percentiles) show the barrier protection (percent) activity of APC on human brain endothelial cells pretreated with recombinant CIDR domains at either 50 μg/ml (dark gray) or 250 μg/ml (light gray) (means and SEM; *n* = 6 from 2 independent experiments done in triplicate). *, *P* < 0.05; **, *P* < 0.01 (by the nonparametric Kruskal-Wallis two-sided test followed by *post hoc* multiple comparisons performed using Dunn’s test).

To study whether *P. reichenowi* CIDR domains interfere with APC binding, competition assays were performed with CHO-EPCR cells. For these assays, CIDR domains were used at 50 μg/ml or 250 μg/ml to achieve approximately 70% and 100% binding levels on CHO745-EPCR cells at the lower and higher concentrations (Fig. S4). As expected, the CD36 binding, *P. reichenowi* var85 CIDRα5 domain did not inhibit APC binding (Fig. 4A and B), whereas the positive-control *P. falciparum* var07 CIDRα1.4 domain almost completely abolished APC binding at 50 μg/ml (85% reduction) or 250 μg/ml (98% reduction). Notably, the three CIDRα1.4 domains from *P. reichenowi* inhibited APC binding by 62% to 85% at the higher concentration (Fig. 4A and B).

10.1128/mSphere.00348-16.5 FIG S4  Flow cytometry titration binding of *P. reichenowi* and *P. falciparum* CIDRα domains to CHO745-EPCR cells. Median levels depicted after normalization to CHO745 background control (*n* = 4). Download FIG S4, PDF file, 0.2 MB. Copyright © 2017 Brazier et al. 2017 Brazier et al. This content is distributed under the terms of the Creative Commons Attribution 4.0 International license.

To study if *P. reichenowi* CIDR domains interfere with APC barrier protective properties, thrombin-induced barrier dysfunction assays were performed with primary human brain microvascular endothelial cells. Thrombin induced a rapid drop in electrical impedance across the brain endothelial monolayer that peaked at approximately 30 min and returned to baseline by 2 h (Fig. 4C). APC diminished thrombin-induced barrier disruption by 50%. As expected, pretreatment with the negative-control CD36 binding *P. reichenowi* var85 CIDRα5 domain led to a minimal reduction in APC barrier protection (25% reduction at the higher concentration) (Fig. 4D). Conversely, the positive-control *P. falciparum* var07 CIDRα1.4 domain and the three CIDRα1.4 domains from *P. reichenowi* caused a 60% to 70% reduction in APC protective function at the higher CIDR concentration. Furthermore, there was a strong concordance between the CIDR-APC competition assay results (Fig. 4B) and blockade of APC protective activity in the barrier permeability assay (Fig. 4D). Thus, *P. reichenowi* and *P. falciparum* CIDRα1.4 domains disrupted the APC-EPCR interaction in similar manners.

**FIG 5:**
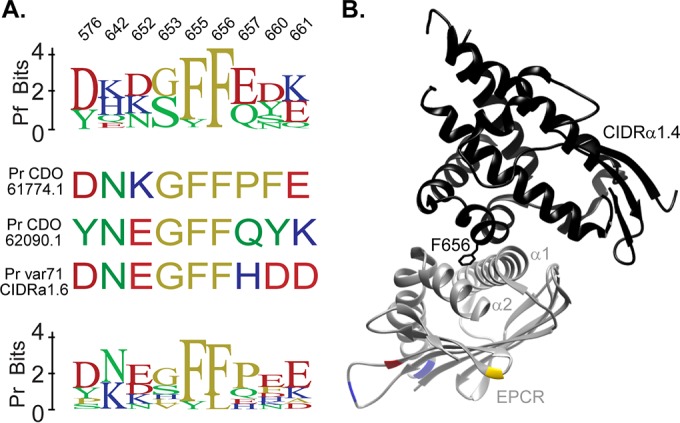
EPCR contact residues are conserved in *P. falciparum* and *P. reichenowi* CIDR sequences. (A) Sequence logos comparing variations between *P. falciparum* (top logo) and *P. reichenowi* (bottom logo) with respect to CIDRα1 residue positions corresponding to those that have direct contact with EPCR in two solved co-crystal structures from *P. falciparum* (26). The residues of the three *P. reichenowi* CIDRα1.4 sequences tested in this study are shown. (B) Structure of the IT4var07 CIDRα1.4-EPCR interaction (26) showing the locations of EPCR residues that differ in gorilla or chimpanzee sequences from human sequences (G124 and I194, gorilla only = blue; S138, chimpanzee only = yellow; A127, gorilla plus chimpanzee = red).

Consistent with the functional analysis, comparison of CIDRα1.4 sequences of *P. falciparum* and *P. reichenowi* shows that they are relatively conserved at nine contact residues from the solved IT4var07 CIDRα1.4-EPCR co-crystal structures (26) (Fig. 5A). This includes the highly conserved dual phenylalanine residues at positions F655 and F656 in IT4var07 CIDRα1.4; the F656 inserts into the EPCR lipid binding groove in a location similar to that of a phenylalanine residue from APC (26) (Fig. 5A). Moreover, chimpanzees and gorillas differ at only 6 amino acid positions from human EPCR (Fig. S5) and all of them are distant from the *P. falciparum* var07 CIDRα1.4-EPCR contact interface (Fig. 5B). Taking the results together, this analysis suggests that *P. reichenowi* and *P. falciparum* CIDRα1.4 domains engage similar surfaces on EPCR and that both compete for binding with its native ligand protein C/APC.

10.1128/mSphere.00348-16.6 FIG S5  Sequence comparison of EPCR from human, chimpanzee, and gorilla. A human EPCR sequence (GenBank accession no. CAG33229.1) is shown at the top of the lineup. Residues that differ in chimpanzee (GenBank accession no. JAA17552.1 and JAA08276.1) and gorilla (GenBank accession no. XP_004062098.1) sequences are indicated. Download FIG S5, PDF file, 0.01 MB. Copyright © 2017 Brazier et al. 2017 Brazier et al. This content is distributed under the terms of the Creative Commons Attribution 4.0 International license.

## DISCUSSION

The uniquely virulent character of the *P. falciparum* parasite, along with its capacity to cytoadhere in the host’s microvasculature, has led to debate regarding the origins of deadly cytoadhesion traits. Several lines of evidence support the hypothesis that widespread human infection with *P. falciparum* is a relatively recent phenomenon and may have emerged in only the past 5,000 to 10,000 years, associated with changes in human populations from hunter-gatherer societies to agriculture-based communities (52). *P. falciparum*’s closest relative is a gorilla parasite, *P. praefalciparum*, and its next closest relative is a chimpanzee parasite, *P. reichenowi* (8). Despite their high genetic similarity to *P. falciparum*, it is unknown whether ape parasites are as virulent in their natural hosts (53). While only limited DNA sequence data exist for *P. praefalciparum*, the genome of *P. reichenowi* has been fully sequenced (42). Here, we investigated the evolutionary origins of *P. falciparum* pathogenicity determinants by studying the binding properties of *P. reichenowi* domains.

For most of the 20th century, the nature of the evolutionary relationship between *P. falciparum* and *P. reichenowi* was largely limited to inferences based upon the morphological similarities between the two parasites (54). These have been extended by genomic studies that show similar catalogs of *var* genes of *P. falciparum* and *P. reichenowi*, as well as of multigene families involved in erythrocyte remodeling (41–44). Although the members of the *var* gene family are among the fastest-evolving genes in *P. falciparum*, there appears to be significant conservation of *var* organization and coding features between the two species (25, 42). In contrast, *P. gaboni*, a more distant *Laverania* relative, contains *var*-like genes that have DBL domains that are highly divergent from those of *P. falciparum* and *P. reichenowi* and lack CIDR domains (41, 42). Taken together, the genomic data suggest an evolutionary history in which DBL domains containing *var*-like genes originated in an early precursor of the *Laverania* subgenus. However, the ancestral *var* gene diverged between *Laverania* species and the characteristic DBL-CIDR head structure of PfEMP1, a key determinant of *P. falciparum* cytoadhesion, is present in only a subset of the members of the *Laverania* clade.

While the origin of the *var* gene family is becoming clearer from comparative genomics, nothing was known about the adhesion traits encoded by *var* genes present in other members of the clade. Extensive work on *P. falciparum* has revealed that the binding properties of CD36 and EPCR are predicted by sequence classification of CIDR domains (26–29). Nevertheless, while phylogenetic classification of PfEMP1 domains is highly predictive of binding (55), exceptions are known. For instance, sequence variation between CIDRα1 domains can determine the ability to bind EPCR (26) or influence the extent of APC blockade activity (30, 38, 40). Here we demonstrated that CIDR domains in *P. reichenowi* can be categorized into sequence types similar to those of *P. falciparum* and that domains bind in a predictable manner to human CD36 and EPCR. More significantly, the CIDRα1 domains from *P. reichenowi* share the capacity of CIDRα1.4 domains from *P. falciparum* to disrupt APC-EPCR binding in a manner that exacerbates the permeability of human brain endothelial cell monolayers in the presence of thrombin (30, 38–40), a feature that may contribute to brain swelling and cerebral malaria pathophysiology (51, 56, 57).

A limitation of this study was that recombinant proteins were analyzed. This was necessary because chimpanzees are an endangered species and because it is technically challenging to work with *P. reichenowi* parasites. However, previous work has shown that recombinant CIDR domains predict *P. falciparum* parasite binding to both CD36 (58) and EPCR (29, 40, 59). These results show that binding to CD36 and EPCR is conserved between *P. falciparum* and the chimpanzee malaria parasite *P. reichenowi*.

The remarkable persistence and specificity of the CD36 and EPCR binding traits in a rapidly evolving variant antigen gene family have several implications. First, they provide evidence that CD36 and EPCR binding have early origins and have been maintained in the populations of both *P. reichenowi* and *P. falciparum* parasites, despite the association of EPCR binding with severe malaria in human infections (29). Consistent with this concept, CD36 and EPCR sequences are highly conserved between humans, chimpanzees, and gorillas. The stability of adhesion receptor specificity suggests a strategy in which the parasite co-opts a key functional molecular interface which the host cannot easily modify without compromising protein function. Indeed, *P. falciparum* CIDRα1 domains interact with the APC binding site in EPCR (26, 29, 30, 39, 40) and CIDRα2-6 domains bind to the oxidized low-density lipoprotein (OxLDL) binding site in CD36 (50). The corresponding EPCR contact residues are completely conserved between human, chimpanzee, and gorilla sequences, and the corresponding CD36 contact residues are identical at 13 of 14 positions in humans and chimpanzees. While gorilla sequences differ at six of the human CD36 contact residues, two of the differences are conservative amino acid substitutions.

An additional implication is that cytoadhesion interactions may have posed less of a barrier than red blood cell invasion (48) for the crossing of the progenitor parasite from gorillas to humans. The ability of the ancestral parasite to cytoadhere to CD36 and EPCR may have been an important factor in the *P. praefalciparum* zoonotic event of transfer to humans, since poor sequestration would otherwise be expected to impose a high parasite fitness cost due to splenic entrapment and clearance of more-rigid IEs (16, 17). Studies of fecal samples have revealed six different *Laverania* species in African chimpanzee and gorilla populations (7–11). It is possible that cytoadhesion interactions may pose a stronger barrier to human transmission for more-distant *Laverania* parasites that lack CIDR domains, but this remains to be determined. Our findings suggest that the adhesion traits encoded in *var* genes may have at least partly preequipped the ancestral *P. falciparum* parasite with traits necessary to survive in the human host. Furthermore, the parasite that crossed into humans was already endowed with the dangerous EPCR binding trait. The long evolutionary persistence of the CIDR-EPCR interaction raises the possibility that it may also possess adaptive properties that have led to its retention in human and chimpanzee parasites, especially since the vast majority of *P. falciparum* infections are not deadly. While CD36 binders are predicted to be more common than EPCR binders, both parasite species have invested considerable genomic resources in retaining both adhesion traits. In addition to OxLDL and APC, a diverse array of ligands bind to CD36 and EPCR (60, 61). This suggests additional possible roles of CD36- and EPCR-based parasite adhesion in influencing a range of physiological and pathological processes which will be of interest to explore.

## MATERIALS AND METHODS

### Sequence analysis.

To identify CIDR domains in *P. reichenowi*, we conducted searches of public databases (http://blast.ncbi.nlm.nih.gov/Blast.cgi?PAGE=Proteins) using amino acid sequences from representatives of the four CIDR sequence types (α, β, γ, and δ) found in the *P. falciparum* genome. This approach identified 93 unique *var* sequences, which were submitted to the VarDom server (http://www.cbs.dtu.dk/services/VarDom/) for the identification of DBL and CIDR domain boundaries. All CIDR domain sequences were extracted, combined with a data set comprising all of the CIDR sequences present in the 3D7 genome, and assembled into a neighbor joining tree using the Geneious Tree Builder tool. For subtyping of *P. reichenowi* CIDRα1 domains, additional representative CIDRα1-8 type domains, as defined by Rask et al. (25), were included in the tree.

### Protein expression.

Recombinant *P. reichenowi* CIDR domains were synthesized as Gblocks gene fragments (Integrated DNA Technologies, Inc.), and *P. falciparum* CIDR sequences were amplified from parasite genomic DNA (gDNA) (see Table S2 in the supplemental material). Proteins were expressed as His6-maltose binding protein (MBP)-tobacco etch virus (TEV)-CIDR-StrepII constructs in pSHuffle expression hosts (New England Biolabs, Inc.) and purified using a two-step process, as described previously (62). Purified proteins were analyzed by SDS/PAGE according to standard procedures.

10.1128/mSphere.00348-16.7 TABLE S2  Sequences of CIDR recombinant proteins. Download TABLE S2, PDF file, 0.04 MB. Copyright © 2017 Brazier et al. 2017 Brazier et al. This content is distributed under the terms of the Creative Commons Attribution 4.0 International license.

### BLI analysis.

CIDR binding kinetics data were determined using an Octet Qke instrument. The protocol for analyzing the CIDR-EPCR interaction was conducted as reported previously (40). The CIDR-CD36 interaction was evaluated by immobilizing hisCD36 to nickel-nitrilotriacetic acid (Ni-NTA) biosensors (ForteBio). For the association phase, binding was measured by immersion of the sensors into wells containing CIDR domains diluted in kinetics buffer (phosphate-buffered saline [PBS], 0.02% Tween-20, 100 μg/ml bovine serum albumin, 0.005% sodium azide) for 600 or 900 s. For the dissociation phase, sensors were then immersed in kinetics buffer for 300 to 600 s. Mean association rate constant (*K*_on_), dissociation rate constant (*K*_off_), and equilibrium dissociation constant (*K*_*d*_) values were calculated using subtracted double-reference data fitted to a 1:1 binding mode using the data analysis software furnished with the Octet instrument (ForteBio).

### Cell binding assays with CIDR recombinant proteins.

To study the binding specificity of CIDR recombinant proteins for CD36 and EPCR, cell binding assays were performed as previously described (40). In brief, stably transfected CHO745-EPCR (40) or CHO745-CD36 (63) cells were lifted with 1× PBS (10 mM EDTA) and resuspended in complete Hanks’ buffered salt solution (HBSS [with 3 mM CaCl_2_, 0.6 mM MgCl_2_, and 1% bovine serum albumin]) to restore divalent cations. For recombinant CIDR binding assays, 1 × 10^5^ cells were incubated with 50 μg/ml CIDR domains for 30 min on ice. In antibody blockade assays, cells were preincubated for 30 min with rat anti-EPCR MAb (RCR-252; Sigma) (50 μg/ml), mouse anti-EPCR MAb 1500 or 1535 (0.2 μM) (64), combined MAbs 1535 and RCR-252, or mouse anti-CD36 MAb FA6-152 (Abcam, Inc.) (20 μg/ml) and then washed two times with 1× HBSS before the CIDR recombinant proteins were added. In CIDR and APC competition binding assays, cells were coincubated for 30 min with 50 μg/ml (660 nM) or 250 μg/ml (3.3 µM) recombinant CIDR domains and 25 μg/ml (446 nM) APC (P2200; Sigma). APC binding was detected with goat anti-human protein C antibody (GAPC-AP; Affinity BioLogicals) (20 μg/ml), followed by chicken anti-goat Alexa488-coupled antibody (A-21467; Molecular Probes) (10 μg/ml). Binding of CIDR recombinant proteins was assessed by labeling with rabbit polyclonal anti-StrepII tag antibody (A00626; GenScript) (10 μg/ml) followed by goat anti-rabbit Alexa488-coupled antibodies (A-11070; Molecular Probes) (4 μg/ml). Labeled cells were analyzed by the use of an LSR II flow cytometer (Becton, Dickinson). Data were analyzed with FlowJo 10 software (Tree Star Inc.).

### Permeability assays.

Endothelial barrier permeability assays were measured in real time using an xCELLigence system from ACEA Biosciences as described previously (30). In brief, primary human brain microvascular endothelial cells (ACBRI376; Cell Systems) were grown for several days to reach confluence in 96-well plates on integrated electronic sensors. For thrombin-induced barrier dysfunction assays, cell monolayers were treated with recombinant CIDR domains (50 or 250 μg/ml) for 30 min, followed by 100 nM APC (Haematologic Technologies, Inc.) for 2 h, followed by 5 nM thrombin. Control wells received thrombin plus thrombin inhibitor hirudin (Hyphen Biomed, France) (500 nM), recombinant CIDR domains alone, APC alone, thrombin alone, or APC plus thrombin treatment. Measurements of transendothelial resistance were initiated before the first treatment and continued until impedance measurements returned to baseline (~2 h after thrombin treatment). CIDR blockade of APC function was measured at the peak of thrombin barrier disruption. Blockade activity was calculated by determining the percentage of APC protection in the presence or absence of CIDR domains.
