# Supplementary figures and images for: Pathogenicity Determinants of the Human Malaria Parasite Plasmodium falciparum Have Ancient Origins
Source: mSphere. 2017 Jan 11;2(1):e00348-16. doi: 10.1128/mSphere.00348-16 (PMC5227068; doi:10.1128/mSphere.00348-16)

**A.**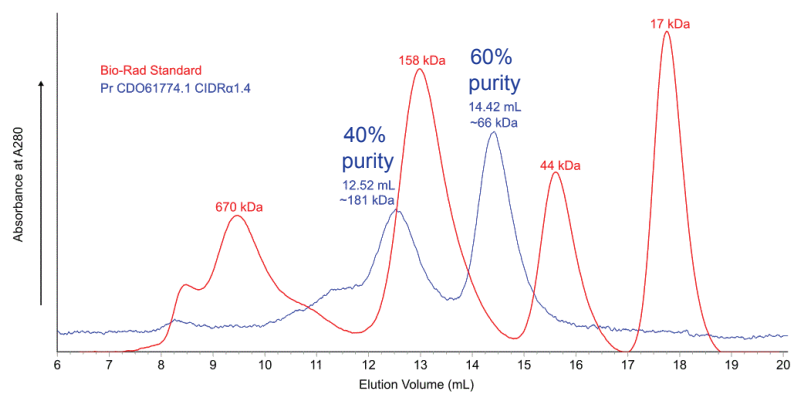**B.**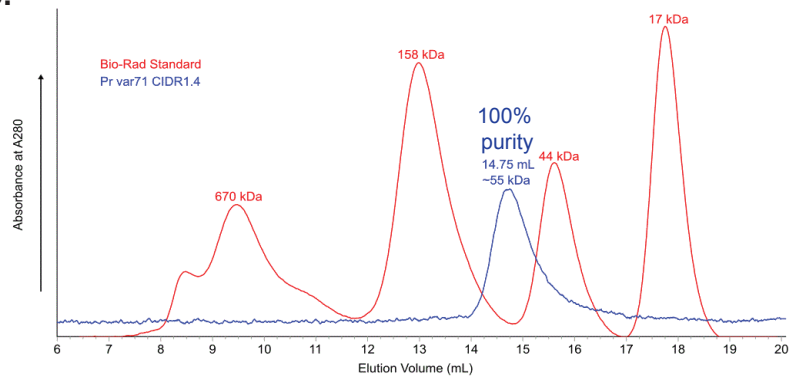**C.**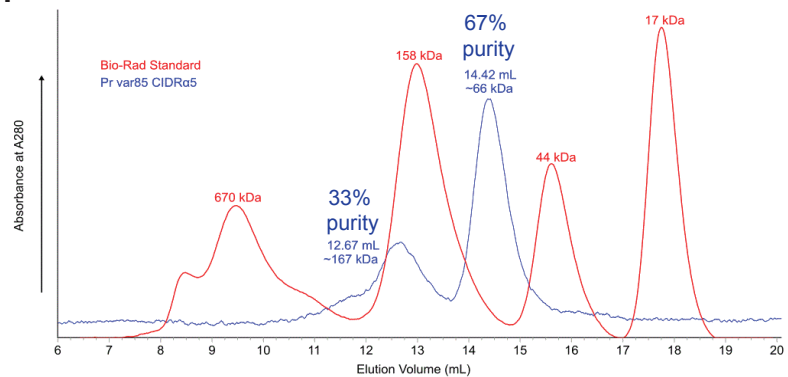

Supplement: FIG S1 [file sph001172221sf1.pdf]

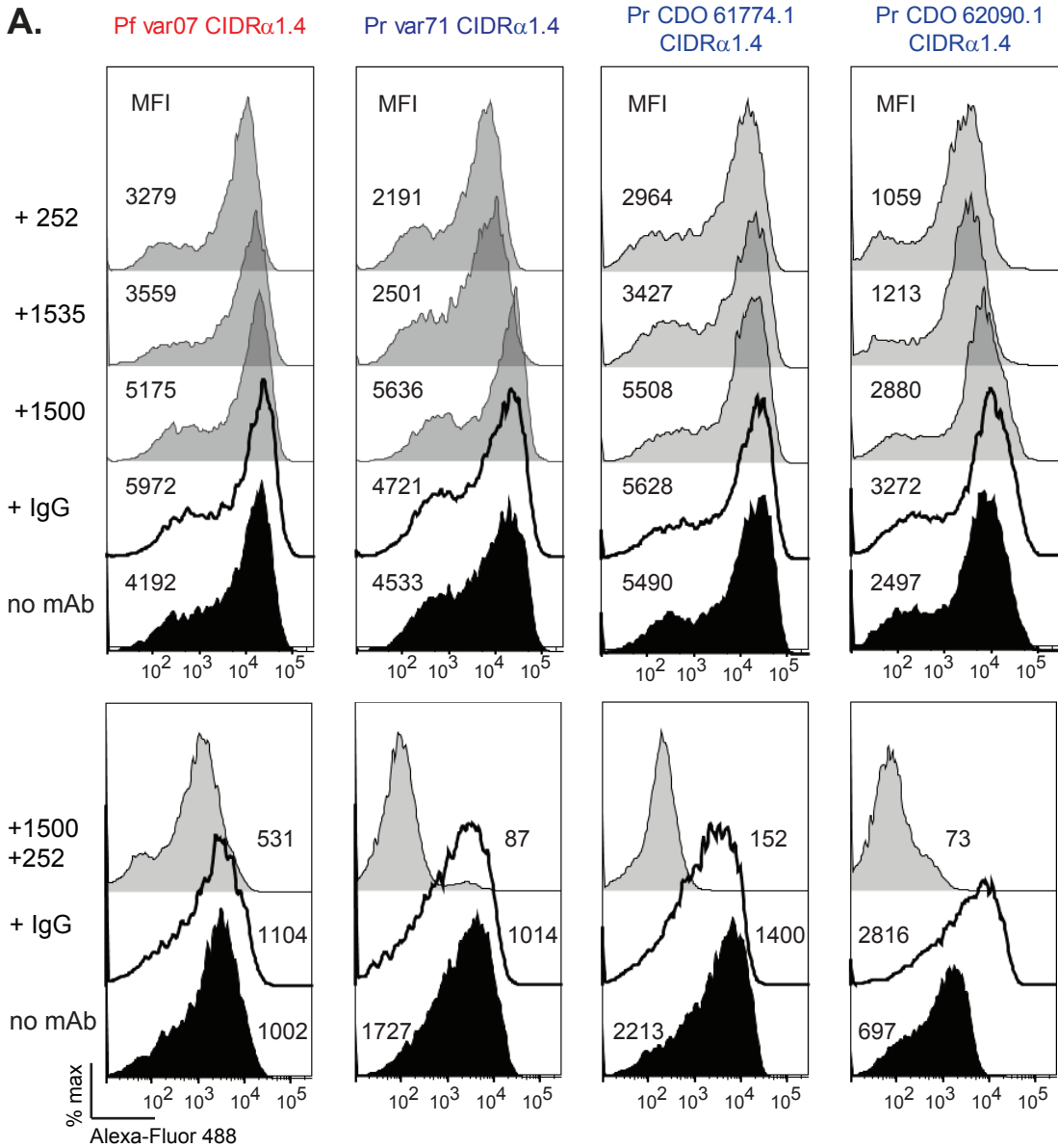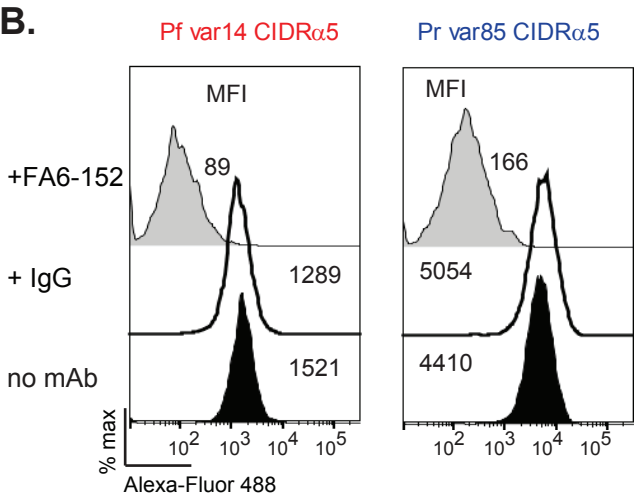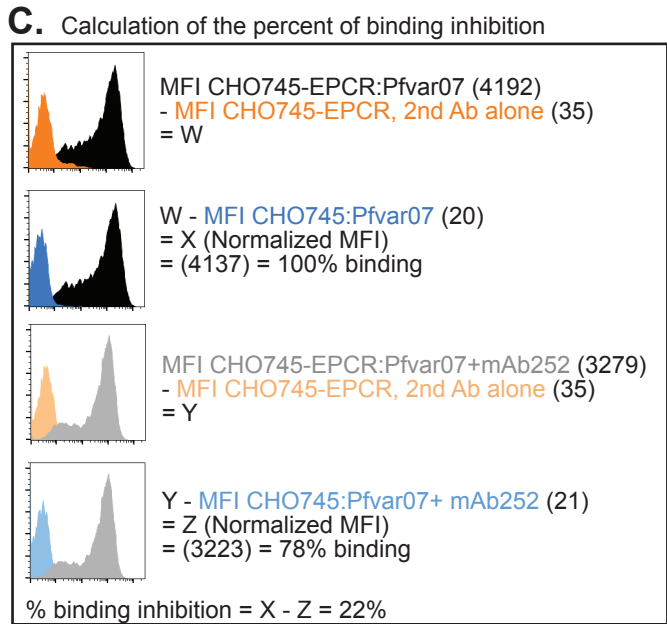

Supplement: FIG S2 [file sph001172221sf2.pdf]

Binding to CHO-745-EPCR

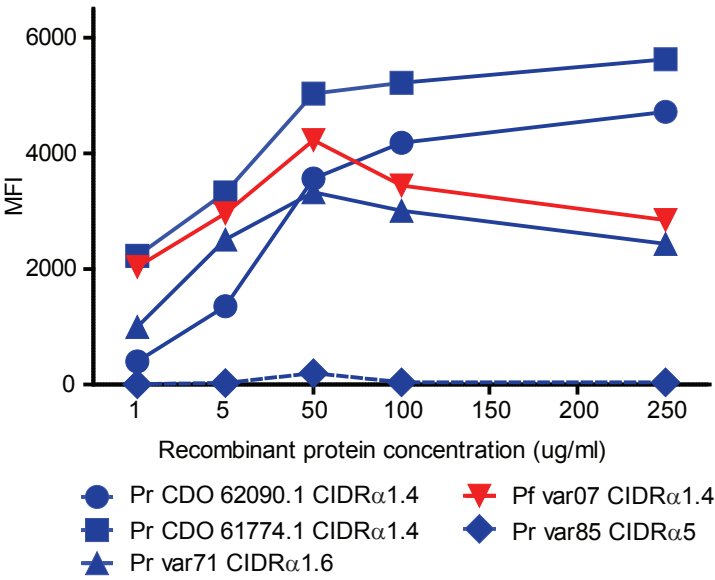

Supplement: FIG S4 [file sph001172221sf4.pdf]
