# Supplementary material for: Pathogenicity Determinants of the Human Malaria Parasite Plasmodium falciparum Have Ancient Origins
Source: mSphere. 2017 Jan 11;2(1):e00348-16. doi: 10.1128/mSphere.00348-16 (PMC5227068; doi:10.1128/mSphere.00348-16)
Supplement: TABLE S1 [file sph001172221st6.pdf]

TABLE S1. Affinities of CIDR domains for EPCR or CD36 as measured by Bio-Layer Interferometry (BLI)

CIDR construct	Subclass	Biosensor	Kd (nM)	k_{on} ($M^{-1}s^{-1}$)	k_{off} (s^{-1})
Pr var71	α1.4	EPCR	23	1.18×10^{4}	2.68×10^{-4}
Pr CD061774.1	α1.4	EPCR	6.2	4.73×10^{4}	2.93×10^{-4}
Pr CDO62090.1	α1.4	EPCR	60	7.35×10^{3}	4.43×10^{-4}
Pr var85	α5	CD36	1.7	1.98×10^{4}	3.42×10^{-5}
Pf var14	α5	CD36	3.2	9.28×10^{3}	2.99×10^{-5}
