# Supplementary material for: Pathogenicity Determinants of the Human Malaria Parasite Plasmodium falciparum Have Ancient Origins
Source: mSphere. 2017 Jan 11;2(1):e00348-16. doi: 10.1128/mSphere.00348-16 (PMC5227068; doi:10.1128/mSphere.00348-16)
Supplement: FIG S5 [file sph001172221sf5.pdf]

```
Human                MLTTLLPILLLSGWAFCSQDASDGLQRLHMLQISYFRDPYHVWYQGNASLGGHLTHVLEGPDTNTTIIQLQPLQEPESWARTQSGLQSYLLQFHGLVRLV
Chimpanzee
Chimpanzee
Gorilla
                     ****************************************************************************************************

Human                HQERTLAFPLTIRCFLGCELPPEGSRAHVFFEVAVNGSSFVSFRPERALWQADTQVTSGVVTFTLQQLNAYNRTRYELREFLEDTCVQYVQKHISAENTK
Chimpanzee                                     P          T                                                          K
Chimpanzee                                     P          T
Gorilla                                     S  P                                                                  T
                     ***********************.**.****:*****:******************************************************* **:***

Human                GSQTSRSYTSLVLGVLVGSFIIAGVAVGIFLCTGGRRC
Chimpanzee                         I
Chimpanzee                         I
Gorilla
                     **************:***********************
```
