# Supplementary material for: Pathogenicity Determinants of the Human Malaria Parasite Plasmodium falciparum Have Ancient Origins
Source: mSphere. 2017 Jan 11;2(1):e00348-16. doi: 10.1128/mSphere.00348-16 (PMC5227068; doi:10.1128/mSphere.00348-16)
Supplement: TABLE S2 [file sph001172221st7.pdf]

TABLE S2: Sequences of CIDR recombinant proteins

His-MBP-Tev-CIDR sequence-Streptactin

MKIHHHHHHEEGKLVIWINGDKGYNGLAEVGKKFEKDTGIKVTVEHPDKLEEKFPQVAATGDG
PDIIFWAHDRFGGYAQSGLLAEITPDKAFQDKLYPFTWDAVRYNGKLIAYPIAVEALSLIYNKDL
LPNPPKTWEEIPALDKELKAKGKSALMFNLQEPYFTWPLIAADGGYAFKYENGKYDIKDVGVD
NAGAKAGLTFLVDLIKNKHMNADTDYSIAEAAFNKGETAMTINGPWAWSNIDTSKVNYGVTVL
PTFKGQPSKPFVGVLSAGINAASPNKELAKEFLENYLLTDEGLEAVNKDKPLGAVALKSYEEELA
KDPRIAATMENAQKGEIMPNIPQMSAFWYAVRTAVINAASGRQTVDEALKDAQTNSITSLYKKA
GLENLYFQG-**CIDR sequence**-WSHPQFEK

Pr CDO61774.1 CIDRα1.4

PHCGVVCNNGTCTDKPNNGNCGNKETYSPGGAKTTEIKVIVSGNEQGDISKKLEDFCSDENNEN
GKNYQKWECYYKSSQNNKCKMETKSGTSITEEKVTSFDAFFDLWVKNLLRDSIKWETELKDCIN
NSNVTDCNNDCNKNCVCFDKWVKQKEEVWKNMKTVLGNQKENLDNYYNKLNGLFKGFFFPV
MFELNHDEAKWNKLMENLKKKIESSKENRGTVNSQDAIELLLEYLKEKSTICKDNNTNEGC

Pr CDO62090.1 CIDRα1.4

PDCGVDCSSGKCEKKEDPDGNCGNKETYNPPPGVPKTKINVLYSGDKQGDITQKLQNFCSDENK
ENGTNYQKWECYYKDEKQNKCKMDKTSGKNMTEDKIMSFDEFFYSWVTNFLIDTINWENELK
NCMNNTILTDCNDGCKINCVCFDKWVKQKENEWENVKKVFENKNGISDNYYNKLKYIFEGFFF
QVLYKLNQDEAKWNQLKQKLKEIIVSSKANTGKEHSQDAIKPLLEYIKEKSTICKDNNTNEGC

Pr var71 CIDRα1.6

PYCGVEYRNGKYNIKKNKDGNCGKNVHYSPPKDVTPIDIHVLYSGEEQVNITKKLENFCTNPND
KHGKNYENWQCYYKDSDDNKCKMTSSSQKEPKHRDVITFHKFFDLWVKNLLKDTIKWESKLK
DCINNTNVTDCNDGCNTNCVCFEKWVKQKEDEWTKVKNVFENKNGISDNYYKKLNDLFEGYF
FHVIKDMYKGEEKWNQLKQKLRTKIDLSKKKKGTNDSEAAIKVLFDHLKETATICKDNNTNEG
C

Pr var85 CIDRα5

PECGVVRNGGTFQKNDEKEGDCSGKNEEYKPPEGVTEHDINFLYSGEGHEDMTKKLSGFCKKPE
DKKGTRNEQWKCYYEKAENNMCKMTNKGANDNEHAKMMSFNDFFNFWVSHLLTDTINWRK
ELNRCIKASKSETCEKKCNKNCKCFQKWIEKKKVEWEEVKDQYDEQKDMDIFNPYWILEYDLE
EQLLPIMEKNNSDLELIGKMKEIIGKNYKNKELKRTDVNAIDILLKHELKDATTCVENDPQENC

Pf var14 CIDRα5

WCGIEEQKDGKWKRINDHSACKEEELYTPKENAKYTKINVLTSGEGHEDIAKRLKEFCTKTQNG
GGGSDDCGGNSDSSLCEPWQCYQPDQLEKVGGGEVDDKLKGAGGLCIFEKMKGEKKVKKQKT
FNNFFNFWVAHVLKDSIDWRTQLTKCLSEDKLKKCEKGCKSNCECFKKWIEKKEKEWIKVKDQ
FNKQTDFLEWKHYLVLETILENYYFENIQKAYGDLKSIQEMKKMIKENKQNKNRTKDDEDALD
VLFDHEKEEAEDCLDIHE

Pf var07 CIDRα1.4

PDCGVICENGKCVVKENGSNCRHYNIYEPAPDVKTTEINVIVSGDEQGIITKKLQDFCMNPNNEN
GTNNQIWKCYYKDEKENKCKVETKSGNSTYKEKITSFDEFFDFWVRKLLIDTIKWETELTYCINN
TTNADCNNECNKNCVCFDKWVKQKEKEWKNIMDLFTNKHDIPKKYYLNINDLFNSFFFQVIYK
FNEGEAKWNKLKENLKKKTESSKKNKGTKDSEAAIKVLFDHLKETATICKDNNTNEAC
